# Efficacy of cecal retroflexion observed on adenoma missing of ascending colon during colonoscopy: A prospective, randomized, pilot trial

**DOI:** 10.1097/MD.0000000000034806

**Published:** 2023-08-25

**Authors:** Cheng-Long Wang, Zi-Ye Zhao, Jia-Yi Wu, Fei-Hu Yan, Jie Yuan, Jun-Jie Xing, Hao Wang, En-Da Yu

**Affiliations:** a Department of Colorectal Surgery and GI Endoscopy Center, Naval Medical University First Affiliated Hospital (Changhai Hospital), Shanghai, China; b Department of Gastroenterology and GI Endoscopy Center, Xiamen University Affiliated Chenggong Hospital (Chinese PLA 73rd Army Corps Hospital), Xiamen, Fujian, China; c Department of Civil Engineering and Architecture, Jinhua Open University, Jinhua, Zhejiang, China.

**Keywords:** adenoma missing, ascending colon, cecal retroflexion observed, colonoscopy

## Abstract

**Background:**

Although colonoscopic retroflexion has been proved effective in reducing missed adenomas, there is still a lack of comprehensive and in-depth research focused on the ascending colon. We aimed to conduct a randomized controlled trial and tandem colonoscopy to investigate whether cecal retroflexion observed during colonoscopy can reduce missed adenomas in the ascending colon.

**Methods:**

Men and women required to be between 45 and 80 years of age were screened for enrollment in the trial. Patients were randomly assigned according to a 1:1 ratio to either the trial group or control group. Patients in the trial group underwent 2 forward examination and a cecal retroflexion observed in the ascending colon, while patients in the control group underwent only 2 forward examinations in the ascending colon. The primary outcome was adenoma miss rate. The secondary outcomes contained adenoma detection rate, polyp miss rate, polyp detection rate, insertion time and withdrawal time. Differences between groups in the primary outcome and in the other categorical indicators were tested using chi-squared test and Fisher exact test. For the comparison of continuous outcomes, the Student *t* test was applied.

**Results:**

A total of 60 subjects were eligible for the study between April to June 2020, of which 55 were randomized and eligible for analysis (26 to the control group and 29 to the trial group). The characteristics of patients were no significant differences statistically between the trial group and the control group. Similarly, the characteristics of the colonoscopy procedures included cecal insertion distance, the length of cecum and ascending colon, insertion time, withdrawal time, quality of bowel preparation, numerical rating scale for pain, polyps detected, and adenomas detected, and there were no significant differences statistically between the 2 groups (*P* = .864, *P* = .754, *P* = .700, *P* = .974, *P* = .585, *P* = .835, *P* = .373, *P* = .489). The characteristics of the polyps were also no significant differences statistically between the 2 groups.

**Conclusion:**

This pilot trial failed to show benefit of cecal retroflexion observed on adenoma missing of ascending colon during colonoscopy; however, further conclusions require a prospective study with a higher level of evidence. (NCT03355443).

## 1. Introduction

Colonoscopic retroflexion is a widely used auxiliary technique for adenoma detection, making the operation more convenient when performing adenoma resection. However, it is usually applied to observe the lower rectum to detect internal hemorrhoids and lesions located in the lower rectum near the anus.^[1]^Weinberg^[2]^ demonstrated that polyp miss rate (PMR) in the ascending colon was significantly higher than that in the distal colon. Additionally, complex lesions in the ascending colon were often located on or behind mucosal folds, making them more difficult to detect through forward colonoscopy.^[3]^ Thus, it is of great clinical value to investigate how to reduce the missed adenomas of ascending colon by using colonoscopic retroflexion, which is still an area of limited and in-depth research. As the performance of colonoscopic devices has improved, retroflexion in the ascending colon has become easier than before. Hewett^[4]^ showed that the success rate of the retroflexion in the ascending colon using Olympus Evis Exera II colonoscopy system was 94.4%, and retroflexion could reduce adenoma miss rate (AMR) by 9.8%, indicating that regular retroflexion in the ascending colon is feasible and effective under conventional equipment conditions. In view of this, the objective of this study was to overcome the defects of most previous studies that mainly use adenoma detection rate (ADR) to evaluate the missed adenomas and the limitation of retrospective data on the colonoscopic retroflexion, and to explore whether cecal retroflexion observed during colonoscopy can reduce missed adenomas of the ascending colon, with using AMR as the primary outcome, through a randomized controlled and tandem colonoscopy trial.

## 2. Methods

### 2.1. Trial design

From April 18, 2020, to June 28, 2020, we conducted a prospective, randomized, patient-blind, controlled, tandem colonoscopy, pilot trial from a single tertiary referral center. Patients who underwent colonoscopy were randomly assigned according to a 1:1 ratio to either a trial group with 2 forward examination and cecal retroflexion observed in the ascending colon or a control group with repeated 2 examination in the ascending colon. AMR and related information were compared between the 2 groups. This trial was undertaken in the gastrointestinal (GI) Endoscopy Center of the Naval Medical University First Affiliated Hospital in Shanghai, China. All the patients were referred from outpatient and inpatient departments of the same medical center. This trail was approved by the Medical Ethics Committee of the Naval Medical University First Affiliated Hospital (EC Approval No.: CHEC2017-142), and all the patients gave written informed consent before colonoscopy. The trial was registered at ClinicalTrials.gov (identifier, NCT03355443). Prof Yu En-Da and other authors had access to the study data and had reviewed and approved the final manuscript. However, there were still some differences between the actual trial and the original study protocol approved by the Medical Ethics Committee. First, our colonoscopy system under retroflexion was not able to visualize all the proximal colon as expected, and second, the timing and sample size of our study did not go as planned due to the impact of COVID-19.

### 2.2. Patient selection

Patients with asymptomatic or nonspecific symptoms in the digestive system (mild abdominal pain, diarrhea, constipation, etc) were screened for enrollment in the trial. All patients were required to be between 45 and 80 years of age. The exclusion criteria were as follows: those under 45 or over 80 years old, family history of colorectal cancer, history of colonoscopy in recent 5 years, history of previous abdominal surgery, pregnancy, uncontrolled hypertension, or involvement in other studies within the past 60 days. (see Supplemental Digital Content A, http://links.lww.com/MD/J570, which illustrates a complete list of the inclusion, exclusion and exit criteria.)

### 2.3. Randomization

Eligible patients (blinded) were randomized to one of the 2 groups in a 1:1 ratio using concealed allocation by a research personnel, with the randomization table being computer generated at the Center for Clinical Epidemiology and Evidence-Based Medicine of Naval Medical University, Shanghai, China. To ensure the integrity of the trial, the research personnel who generated the randomization table was not involved in the colonoscopic procedure. However, due to the nature of the intervention, both the colonoscopist and research personnel were aware of the trial group assignments after randomization.

### 2.4. Trial interventions

For patients assigned to the control group, after cecal intubation, the colonoscopist performed 2 forward examinations from the cecum to the hepatic flexure during withdrawal, recording the examination distance (cecum insertion distance and hepatic flexure insertion distance), examination time (total examination time, cecum insertion time, first withdrawal time from cecum to hepatic flexure, second withdrawal time from cecum to hepatic flexure, total withdrawal time), polyps detection (location and size of polyps found from cecum to hepatic flexure for the first and second time), numerical rating scale for pain (NRS), and any complications.

For patients assigned to the trial group, after cecal intubation, the colonoscopist first performed a forward examination from the cecum to the hepatic flexure during withdrawal, then reentered the cecum and performed a reverse observation using colonoscopic retroflexion, before continuing to withdraw the colonoscope to the hepatic flexure with the forward examination and recorded the same information as the control group.

The examination speed of cecal retroflexion observed was performed according to the routine examination, with the specific examination time determined based on the lesion conditions, and no mandatory requirement. If colonoscopic retroflexion was difficult or unsuccessful after 15 minutes, the attempt should be stopped, and the colonoscopy should be continued, with the sample being exited from the group and not included in the study. Furthermore, no special requirements were applied for other auxiliary techniques (such as abdominal compression and body position change) except for cecal retroflexion observed.

### 2.5. Data collection

When patients were recruited, they signed the informed consent form in outpatient setting, and research personnel collected the patients information and filled out the form, including outpatient category, name, ID number, gender, age, contact phone number, intestinal laxatives, main symptoms, and comments, etc. The colonoscopy was performed by Prof Yu En-Da, a renowned colonoscopy specialist in China, with the assistance of experienced nurses (colonoscopy assistants) from the GI Endoscopy Center. The colonoscopy report was completed by Prof Yu En-Da, mainly noting the quality of bowel preparation (QBP), whether the cecum was intubated, and whether the ileocecal valve and appendiceal fossa were observed. Meanwhile, the number, size, location, and pathological type of polyps, as well as the examination time, were recorded. All the polyps resected were processed and analyzed by pathologists, and the pathological results were issued by the same medical center.

### 2.6. Sample size

We aimed to provide prospective high-quality data on whether cecal retroflexion observed during colonoscopy can reduce missed adenomas in the ascending colon, as no retrospective reports had suggested this. However, due to the exceptional circumstances of a China and even global pandemic collapsing hospital care, this randomized controlled trial (RCT) was conducted without a sample size calculation based on expectations of statistically significant differences. Nonetheless, the final sample size does not affect the significance of the trial’s scientific impact.

### 2.7. Outcomes and statistical analysis

The primary outcome was AMR, the calculation was performed as follows:


$$\begin{array}{l} AMR=\frac{The\;number\;of\;missed\;adenomas}{The\;total\;number\;of\;adenomas} \end{array}$$

$$\begin{array}{l} =\;\;\frac{The\;number\;of\;new\;adenomas\;detected\;by\;a\;second\;colonoscopy}{\begin{array}{lll} & The\;number\;of\;adenomas\;detected\;by\;the\;first\;colonoscopy\; & +The\;number\;of\;new\;adenomas\;detected\;by\;a\;second\;colonoscopy\; \end{array}} \end{array}$$

The secondary outcomes were ADR, PMR, polyp detection rate (PDR), insertion time (IT) and withdrawal time (WT).AMR, ADR, PMR, and PDR were calculated in each group, and then subgroup analysis was performed according to the characteristics of patients (age, gender, indications, type of laxatives, and time period of examination), the characteristics of colonoscopy procedures (cecal insertion distance, the length of cecum and ascending colon, IT, WT, QBP, NRS, polyps detected, and adenomas detected) and the characteristics of polyps (the first detected and missed polyps, the first detected and missed adenomas).(see Supplemental Digital Content B, http://links.lww.com/MD/J571, which demonstrates more definitions and illustrations of correlation indicators.)

Categorical data are presented as number and percentage and compared by *χ^2^* test. If the theoretical frequency T < 1 or the total number of cases n < 40 exist in the 4 fold table data, *Fisher* exact method is employed. Continuous variables are expressed as median and ranges or mean ± standard deviation and compared with the Student *t* test. Two-tailed *P* values < .05 were considered statistically significant, and all statistical analysis of the data was performed with Microsoft Excel (Microsoft Corp., Redmond, WA), and SPSS Statistics 19 (IBM, Armonk, NY).

## 3. Results

### 3.1. Patients

**Figure 1.**
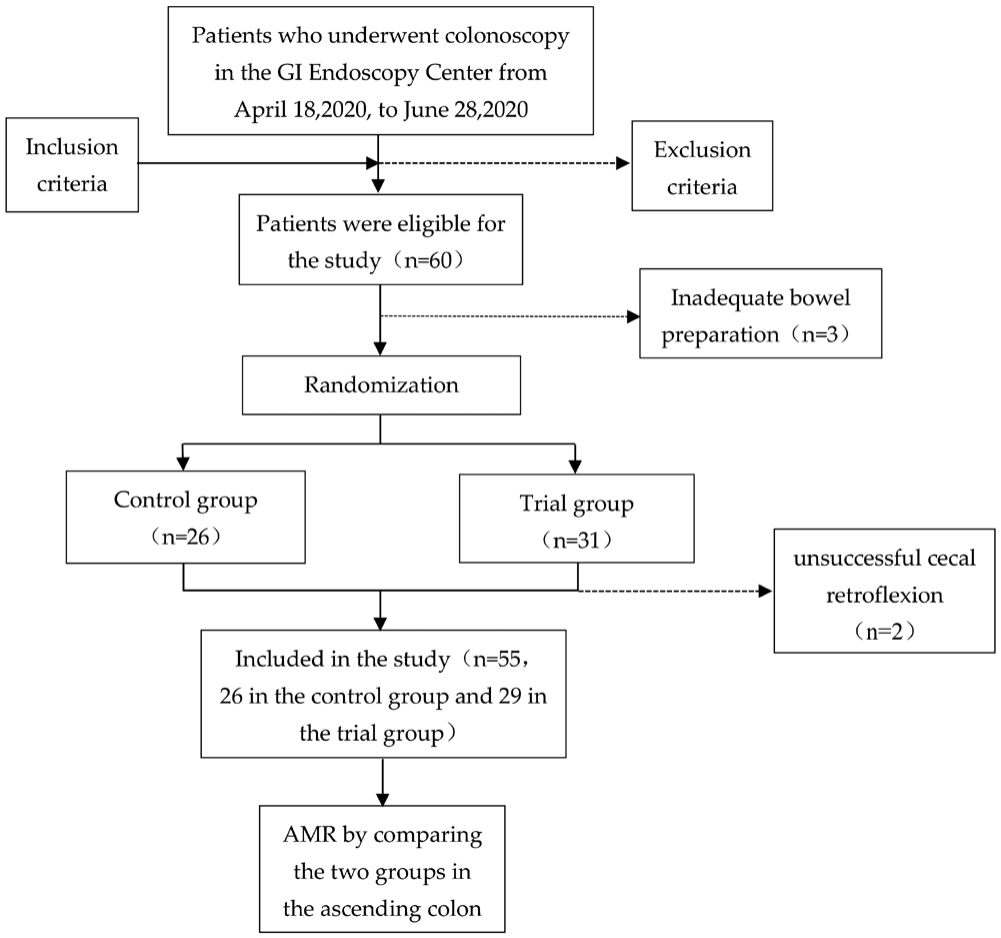
Technical roadmap of the patients.

From April 18, 2020, to June 28, 2020, a total of 60 patients were eligible for the study following the screening of inclusion and exclusion criteria. Of these, 3 patients were exited due to inadequate bowel preparation, and 57 patients were randomized (26 in the control group and 31 in the trial group). During the trial, 2 patients were found to have unsuccessful cecal retroflexion and were exited from the group. Finally, 55 patients completed the trial and were included in the study (26 in the control group and 29 in the trial group) (Fig. 1). No serious complication occurred in any patient during 1 month of follow-up, and the cecal intubation rate was 100%.

### 3.2. Characteristics of patients between the 2 groups

The mean age of the control group was higher than that of the trial group, but there was no statistically significant difference between the 2 groups [(57.27 ± 7.94) years vs (53.48 ± 7.96) years, *P* = .084]; furthermore, there was no significant difference in the gender (female or male), indications (screening or diagnosis), the proportion of patients who used laxatives of MgSO_4_ or Heshuang and the proportion of patients examined in the morning or afternoon between the 2 groups (*P* = .680, *P* = .227, *P* = .247, and *P* = .128, respectively). (Details are presented in Table 1.)

**Table 1:** Characteristics of patients.

Characteristics	Control group	Trial group	Statistical quantity	*P* value
	(n = 26)	(n = 29)		
Age*(yr)	57.27 ± 7.94	53.48 ± 7.96	1.764†	.084
Gender			0.170	.680
Female	12	15		
Male	14	14		
Indications			1.460	.227
Screening	15	12		
Diagnosis	11	17		
Type of laxatives			1.340	.247
MgSO_4_	17	23		
Heshuang	9	6		
Time period of examination			2.316	.128
Morning	18	25		
Afternoon	8	4		

* Mean ± SD.

† Age is the *t* value, and the rest are the *χ^2^* value.

### 3.3. Characteristics of colonoscopy procedures between the 2 groups

The mean distance of cecal insertion in the control group was higher than that in the trial group, but there was no significant difference between the 2 groups [(78.85 ± 8.16) cm vs (78.48 ± 7.52) cm, *P* = .864]; the mean length of cecum and ascending colon in the control group was not significantly different from that in the trial group [(14.42 ± 3.37) cm vs (14.76 ± 4.38) cm, *P* = .754]; there was no significant difference in the mean IT between the 2 groups [(366.00 ± 251.86) s vs (344.10 ± 161.62) s, *P* = .700]; there was no significant difference in the mean WT between the two groups [(344.31 ± 133.72) s vs (343.00 ± 163.11) s, *P*= .974]; there was no significant difference in the mean QBP score between the 2 groups [(2.42 ± 0.86)m vs (2.31 ± 0.66)m, *P* = .585];there was no significant difference in the mean NRS between the 2 groups [(4.19 ± 2.83)p vs (4.03 ± 2.77)p, *P* = .835]; the number of positive patients with polyps detected in the control group and trial group was 6 and 4, respectively, resulting in PDRs of 23.1% (6/26) and 13.8% (4/29) with no statistically significant difference (*P* = .373); adenoma was detected in 2 patients and 1 patient in the control group and trial group, respectively, resulting in ADRs of 7.7%(2/26) and 3.4%(1/29) with no statistically significant difference(*P* = .489). (Details are presented in Table 2.)

**Table 2:** Characteristics of colonoscopy procedures.

Characteristics	Control group	Trial group	Statistical quantity	*P* value
	(n = 26)	(n = 29)		
Cecal insertion distance*(Centimeter)	78.85 ± 8.16	78.48 ± 7.52	0.172	.864
The length of cecum and ascending colon*(Centimeter)	14.42 ± 3.37	14.76 ± 4.38	−0.316	.754
IT*(Second)	366.00 ± 251.86	344.10 ± 161.62	0.388	.700
WT*(Second)	344.31 ± 133.72	343.00 ± 163.11	0.032	.974
QBP*(Point)	2.42 ± 0.86	2.31 ± 0.66	0.550	.585
NRS*(Point)	4.19 ± 2.83	4.03 ± 2.77	0.209	.835
Polyp detected			0.794†	.373
Positive	6	4		
Negative	20	25		
Adenoma detected			0.479†	.489
Positive	2	1		
Negative	24	28		

IT = insertion time, NRS = numerical rating scale for pain, QBP = quality of bowel preparation, WT = withdrawal time.

* Mean ± SD.

† Polyp and adenoma detected is the *χ*^2^ value, and the rest are the *t* value.

### 3.4. Characteristics of polyps between the 2 groups

In the control group, 10 polyps were detected, of which 5 were detected for the first time and 5 were missed for the first time, resulting in a PMR of 50% (5/10). In the trial group, 5 polyps were detected, all of which were detected for the first time, resulting in a PMR of 0% (0/5). However, the difference in PMR between the 2 groups was not statistically significant (*P* = .101). Additionally, in the control group, 4 adenomas were detected, including 2 adenomas detected for the first time and 2 adenomas missed for the first time, resulting in an AMR of 50% (2/4). In the trial group 1 adenoma was detected, which was detected for the first time, resulting in an AMR of 0% (0/1). Again, there was no statistically significant difference between the 2 groups (*P* = 1.000). (Details are presented in Table 3.)

**Table 3:** Characteristics of polyps.

Characteristics	Group		*P* value
	Control group	Trial group	
Polyps	10	5	0.101*
Detected for the first time	5	5	
Missed for the first time	5	0	
Adenomas	4	1	1.000*
Detected for the first time	2	1	
Missed for the first time	2	0	

* *Fisher* exact method.

## 4. Discussion

Colonoscopic retroflexion is an auxiliary examination technique widely used in clinical practice at present, mainly to observe the lower rectum and conveniently diagnose and treat internal hemorrhoids and lesions located near the anus. However, studies have shown that PMR in colonoscopy is as high as 22%,^[5]^ with a significantly higher PMR in the ascending colon compared to the distal colon,^[2]^ making it an urgent concern for scholars to find ways to reduce the missed adenomas of ascending colon through colonoscopic retroflexion. Saad and Rex^[6]^ were the first to study the impact of conventional colonoscopic retroflexion on PDR in 2006, with the results showing no significant effect. However, this study focused on the retroflexion of the rectum, which was not consistent with the main missed area, thus limiting its reference value. Pickhardt study^[3]^ of the missed area revealed that 14 of 15 nonrectal adenomas (93.3%) were located on the folds, with 10 (71.4%) located on the back of the folds, making it difficult to turn and retroflex When the colonoscope was inserted into the ascending colon. Therefore, to address the practical difficulties of conventional colonoscopic retroflexion in the ascending colon, auxiliary equipment was used in earlier studies. Aventis Medical ‘s TEC, a “3-eye” rearview endoscope system, was used to perform retroflexion examination during withdrawal. Researchers have conducted a series of studies on the safety and effectiveness of this technology,^[7,8]^with Waye^[9]^ finding that TEC detected an additional 13.2% of polyps and 11% of adenomas in a single-group tandem trial, and was more effective in the ascending colon due to its ability to detect lesions hidden behind folds. Leufkens^[10]^ also demonstrated that TEC could significantly reduce the PMR through randomized controlled tandem study. A subsequent analysis by Siersema^[11]^ also showed that TEC was more effective in reducing missed lesions in polyp surveillance patients and diagnostic examination patients than in screening patients. These results confirm the value of retroflexion, but TEC cannot be widely used in the screening of colorectal cancer due to its high cost.

Researchers began to focus again on how to cost-effectively perform retroflexion to improve screening efficacy with conventional colonoscopy, as the performance of colonoscopic devices had improved, making the compliance of the colonoscope better and retroflexion easier than before. The study by Hewett^[4]^ showed that the success rate of retroflexion in the ascending colon using Olympus Evis Exera II colonoscopy system was 94.4%, with the loop of the colonoscopic body being the main cause of unsuccessful retroflexion, and that colonoscopic retroflexion could reduce AMR by 9.8%. In 2015 and 2016, 4 studies investigated the significance of colonoscopic retroflexion in the ascending colon with the success rate of retroflexion being 95.9%,^[12]^ 82.4%,^[13]^ 92%^[14]^ and 93.5%,^[15]^ respectively. A meta-analysis of Cohen^[16]^ showed that 8 studies included 3660 subjects, with a total success rate of retroflexion of 91.9%, a reduction of 16.9% in AMR compared with conventional colonoscopy, and a complication rate of 0.03%. (Details are presented in Table 4.) This suggests that, with conventional colonoscopy, colonoscopic retroflexion in the ascending colon is both feasible and effective when performed appropriately.

**Table 4:** Previously reported data of colonoscopic retroflexion in the ascending colon.

Researcher	Country	Number of centers	Design	Type of colonoscopy	Number of subjects	success rate of retroflexion (%)	PMR, %	AMR, %
Hewett, 2011^[4]^	USA	2	Tandem	Evis ExeraII	1000	94.4	9.7	9.8
Chandran, 2015^[12]^	Australia	3	Tandem	CF-H180/190	1351	95.9	11.6	12.4
Lee, 2017^[13]^	Korea	Single	Tandem	CF-H260AL	1020	82.4	9.18	10.4
Triantafyllou, 2016^[14]^	Greece	Single	Tandem	CF-Q145L	674	92	4.96	5.1
Kushnir, 2015^[15]^	USA	2	Tandem, RCT	CF180/190	450	93.5	Nr	18.9
Cohen, 2017^[16]^	Na	8	Meta-analysis	Nr	3660	91.9	Nr	16.9

AMR = adenoma miss rate, Na = not applicable, Nr = not reported, RCT = randomized controlled trial, PMR = polyp miss rate.

However, Rex found that colonoscopic retroflexion in the second withdrawal of the ascending colon could not significantly reduce the miss rate of lesion. Meanwhile, the relevant literature did not recommend the use of colonoscopic retroflexion in the ascending colon during conventional colonoscopy due to its lack obvious benefits and its high operational risks, which may increase the risk of intestinal perforation.^[4,17,18]^

Although the results of colonoscopic retroflexion in the ascending colon were mixed in previous relevant studies from several years ago, the conventional colonoscopic devices and operational performance have been continuously updated. It has been reported that due to the wide lumen diameter of the ascending colon, experienced colonoscopists are usually able to perform the retroflexion of conventional colonoscopy in the ascending colon.^[18]^Therefore, this trial was based on the GI Endoscopy Center of Naval Medical University First Affiliated Hospital, Shanghai, China, which has advanced colonoscopic devices and abundant sources of patients. In addition, Prof Yu En-Da, with first-class colonoscopy skills in China, was invited to personally operate colonoscopy for each enrolled patient. However, given the lack of previous experience of colonoscopic retroflexion in ascending colon and potential operation risks, we first selected the cecum, which was wider and easier to operate in the ascending colon, as the retroflexion position to preliminarily explore whether cecal retroflexion observed in colonoscopy could reduce missed adenomas of the ascending colon.

**Figure 2.**
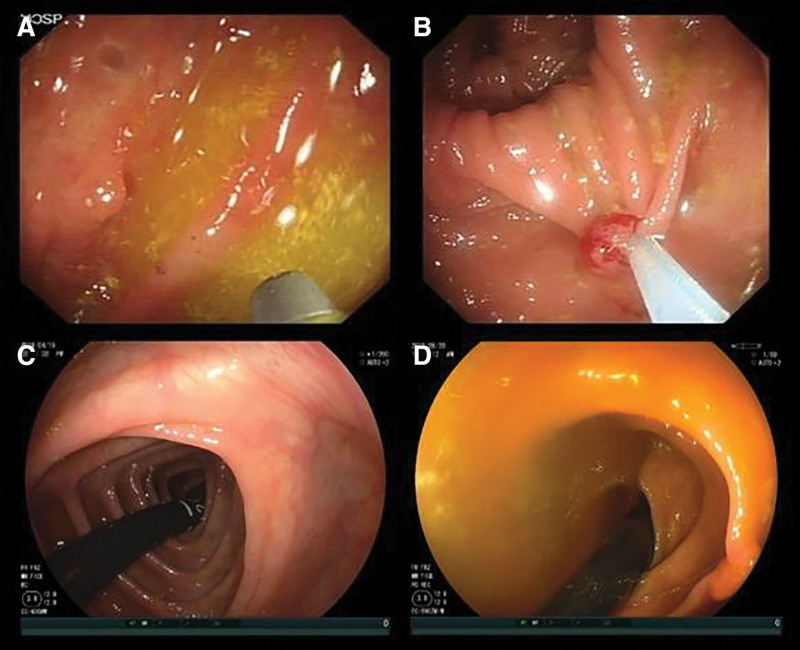
Colonoscopic images taken in the trial. (A) Moderate quality of bowel preparation (QBP) during forward examination, (B) Good QBP during forward examination, (C) excellent QBP under cecal retroflexion observed, and (D) poor QBP under cecal retroflexion observed.

The results of this trial showed that there was no statistically significant difference between the 2 group, indicating that cecal retroflexion observed during colonoscopy may not have much significance for adenoma missing in the ascending colon. The reasons for this could be attributed to the following: The trial period was hampered by the heavy impact of COVID-19 in China, resulting in an insufficient sample size. The evidence level from the preliminary report is limited, but it can be used as a reference for RCTs with sufficient sample size in our ongoing study post-pandemic; Even under moderate QBP, polyps could be found and resected in the visual field during forward examination; however, under cecal retroflexion observed, even with excellent QBP, polyps could only be observed in the 2 or 3 colonic folds closest to the cecum, making it difficult to identify polyps in the far ascending colon, particularly under poor QBP (Fig 2.).Therefore, cecal retroflexion observed has a limited visual field, which affects the observation of ascending colon and the detection of missed adeomas; The colonoscopic device used in this study (Olympus colonoscope CF-Q260 AI; Olympus, Tokyo, Japan) was not consistent with that used in previous studies of successful colonoscopic retroflexion in the ascending colon (Table 4), which may have affected the results of this study to some extent; This trial was only patient-blind, so subjective evaluations from the colonoscopist, research personnel and colonoscopy assistants may have produce some bias to the results.

Of course, our trial is only a preliminary exploration at present, but in terms of clinical significance, it not only tests the scientificity and feasibility of the trial design, but also provides a reference for further RCTs in our next study. Moreover, it also takes a meaningful step towards quality control of colonoscopy. We believe that prospective studies with higher evidence levels will surely bring us more valuable information in the future.

## 5. Conclusion

This RCT demonstrated that tandem colonoscopy with a “cecal retroflexion observed” intervention could not detect more missed adenomas and reduce AMR of the ascending colon compared to conventional tandem colonoscopy. Consequently, cecal retroflexion observed during colonoscopy may not have much significance for adenoma missing in the ascending colon; however, further conclusions require a prospective study with a higher level of evidence.

## Acknowledgments

We thank the patients for their participation. We appreciate very much for all the medical staffs of Department of Colorectal Surgery and GI Endoscopy Center and pathologists in Naval Medical University First Affiliated Hospital for their hard work and administrative support of Chinese PLA 73rd Army Corps Hospital.

## Author contributions

**Conceptualization:** Cheng-Long Wang, Zi-Ye Zhao.

**Data curation:** Cheng-Long Wang, Zi-Ye Zhao.

**Formal analysis:** Cheng-Long Wang, Zi-Ye Zhao.

**Funding acquisition:** Fei-Hu Yan, En-Da Yu.

**Investigation:** Cheng-Long Wang, Zi-Ye Zhao, Fei-Hu Yan, Jie Yuan.

**Methodology:** Zi-Ye Zhao, Jia-Yi Wu.

**Project administration:** En-Da Yu.

**Resources:** Cheng-Long Wang, Zi-Ye Zhao, Fei-Hu Yan, Jie Yuan.

**Software:** Cheng-Long Wang, Jia-Yi Wu.

**Supervision:** Jun-Jie Xing, Hao Wang, En-Da Yu.

**Validation:** Cheng-Long Wang, Jun-Jie Xing, Hao Wang.

**Visualization:** Cheng-Long Wang, Jia-Yi Wu.

**Writing – original draft:** Cheng-Long Wang.

**Writing – review & editing:** Jun-Jie Xing, Hao Wang, En-Da Yu.

## Supplementary Material





## References

[1] Floer M Meister T. Endoscopic improvement of the adenoma detection rate during colonoscopy - where do we stand in 2015. Digestion. 2016;93:202–13. 26986225 10.1159/000442464

[2] Weinberg DS. Colonoscopy: what does it take to get it “right.”. Ann Intern Med. 2011;154:68–9. 21200044 10.7326/0003-4819-154-1-201101040-00013

[3] Pickhardt PJ Nugent PA Mysliwiec PA. Location of adenomas missed by optical colonoscopy. Ann Intern Med. 2004;141:352–9. 15353426 10.7326/0003-4819-141-5-200409070-00009

[4] Hewett DG Rex DK. Miss rate of right-sided colon examination during colonoscopy defined by retroflexion: an observational study. Gastrointest Endosc. 2011;74:246–52. 21679946 10.1016/j.gie.2011.04.005

[5] van Rijn JC Reitsma JB Stoker J. Polyp miss rate determined by tandem colonoscopy: a systematic review. Am J Gastroenterol. 2006;101:343–50. 16454841 10.1111/j.1572-0241.2006.00390.x

[6] Saad A Rex DK. Routine rectal retroflexion during colonoscopy has a low yield for neoplasia. World J Gastroenterol. 2008;14:6503–5. 19030202 10.3748/wjg.14.6503 PMC2773336

[7] Triadafilopoulos G Li J. A pilot study to assess the safety and efficacy of the third eye retrograde auxiliary imaging system during colonoscopy. Endoscopy. 2008;40:478–82. 18543136 10.1055/s-2007-995811

[8] DeMarco DC Odstrcil E Lara LF. Impact of experience with a retrograde-viewing device on adenoma detection rates and withdrawal times during colonoscopy: the third eye retroscope study group. Gastrointest Endosc. 2010;71:542–50. 20189513 10.1016/j.gie.2009.12.021

[9] Waye JD Heigh RI Fleischer DE. A retrograde-viewing device improves detection of adenomas in the colon: a prospective efficacy evaluation (with videos). Gastrointest Endosc. 2010;71:551–6. 20018280 10.1016/j.gie.2009.09.043

[10] Leufkens AM DeMarco DC Rastogi A. Effect of a retrograde-viewing device on adenoma detection rate during colonoscopy: the TERRACE study. Gastrointest Endosc. 2011;73:480–9. 21067735 10.1016/j.gie.2010.09.004

[11] Siersema PD Rastogi A Leufkens AM. Retrograde-viewing device improves adenoma detection rate in colonoscopies for surveillance and diagnostic workup. World J Gastroenterol. 2012;18:3400–8. 22807609 10.3748/wjg.v18.i26.3400 PMC3396192

[12] Chandran S Parker F Vaughan R. Right-sided adenoma detection with retroflexion versus forward-view colonoscopy. Gastrointest Endosc. 2015;81:608–13. 25440687 10.1016/j.gie.2014.08.039

[13] Lee HS Jeon SW Park HY. Improved detection of right colon adenomas with additional retroflexion following two forward-view examinations: a prospective study. Endoscopy. 2017;49:334–41. 27931050 10.1055/s-0042-119401

[14] Triantafyllou K Tziatzios G Sioulas AD. Diagnostic yield of scope retroflexion in the right colon: a prospective cohort study. Dig Liver Dis. 2016;48:176–81. 26748425 10.1016/j.dld.2015.11.024

[15] Kushnir VM Oh YS Hollander T. Impact of retroflexion vs. second forward view examination of the right colon on adenoma detection: a comparison study. Am J Gastroenterol. 2015;110:415–22. 25732415 10.1038/ajg.2015.21 PMC4535185

[16] Cohen J Grunwald D Grossberg LB. The effect of right colon retroflexion on adenoma detection: a systematic review and meta-analysis. J Clin Gastroenterol. 2017;51:818–24. 27683963 10.1097/MCG.0000000000000695 PMC5373924

[17] Harrison M Singh N Rex DK. Impact of proximal colon retroflexion on adenoma miss rates. Am J Gastroenterol. 2004;99:519–22. 15056095 10.1111/j.1572-0241.2004.04070.x

[18] Wanders LK van Doorn SC Fockens P. Quality of colonoscopy and advances in detection of colorectal lesions: a current overview. Expert Rev Gastroenterol Hepatol. 2015;9:417–30. 25467213 10.1586/17474124.2015.972940

