# Supplementary material for: Efficacy of cecal retroflexion observed on adenoma missing of ascending colon during colonoscopy: A prospective, randomized, pilot trial
Source: Medicine (Baltimore). 2023 Aug 25;102(34):e34806. doi: 10.1097/MD.0000000000034806 (PMC10470795; doi:10.1097/MD.0000000000034806)
Supplement: Supplementary file 1 [file medi-102-e34806-s001.pdf]

Appendix A

Inclusion criteria: 1) The patients who underwent colonoscopy were admitted to the outpatient and inpatient of the whole hospital; 2) Age ⩾45 years and ⩽80 years; 3) Asymptomatic or nonspecific symptoms such as mild abdominal pain, diarrhea, constipation, etc.; 4) Be able to understand the purpose of the trial and sign the informed consent form.

Exclusion criteria: 1) Family history of colorectal cancer (CRC) in immediate family members, history of familial hereditary adenomatous polyposis in immediate family members under 60 years old, mainly including familial adenomatous polyposis, juvenile polyposis and hereditary nonpolyposis CRC; 2) History of various digestive system tumors including CRC, inflammatory bowel disease, etc.; 3) Colorectal polyps, tumor-related symptoms such as long-term melena, fine stool, etc.; 4) History of colonoscopy in recent 5 years; 5) History of abdominal and pelvic surgery and radiotherapy; 6) Uncontrolled hypertension (untreated systolic blood pressure>160mmHg, or diastolic blood pressure>95mmHg); 7) History of stroke, coronary artery disease, or vascular disease; 8) pregnancy; 9) refusal to undergo conscious colonoscopy; 10）Involvement in other trails in the last 60 days before this trial.

Exit criteria: 1) Patients whose QBP cannot meet the trial needs; 2) Patients with unsuccessful cecal retroflexion under colonoscopy; 3) Patients with bleeding, perforation, and other complications; 4) Refusal to undergo regular colonoscopy for various personal reasons.
