# Supplementary material for: Efficacy of cecal retroflexion observed on adenoma missing of ascending colon during colonoscopy: A prospective, randomized, pilot trial
Source: Medicine (Baltimore). 2023 Aug 25;102(34):e34806. doi: 10.1097/MD.0000000000034806 (PMC10470795; doi:10.1097/MD.0000000000034806)
Supplement: Supplementary file 2 [file medi-102-e34806-s002.pdf]

Appendix B

Definitions and instructions

1. Bowel preparation: Patients underwent a low residue diet the day before colonoscopy and took 2 bags of compound polyethylene glycol electrolyte powder II (Heshuang, Wanhe, Shenzhen, China) or 50 g $MgSO_4$ in the morning of colonoscopy, with an appropriate dose added in the evening before colonoscopy for patients with previous constipation. Bowel preparation was scored by the Aronchick scale, and according to the Aronchick scale, the QBP was evaluated as "excellent", "good", "moderate", "poor" or "inadequate"[19]. In this study, the scores were divided into five grades, which were respectively recorded as "excellent" (4 points),"good" (3 points),"moderate" (2 points),"poor" (1 point) and "inadequate" (0 point). "inadequate" scores were exited from this study.

2. NRS: Patients in this study will mark a number from 0 to 10 according to their personal perception and level of pain after completing colonoscopy, with 0 indicating no pain and 10 indicating the most severe pain. The specific pain levels are divided into four categories: 0 for no pain, 1-3 for mild pain, 4-6 for moderate pain, and 7-10 for severe pain.

3. Others: 1) ADR is the proportion of patients with adenomas detected in all patients. 2) PMR is the proportion of polyps missed in the first colonoscopy in the total number of polyps detected in tandem colonoscopy. 3) PDR is proportion of patents with polyps detected in all patients.
